# Blockade of Indoleamine 2, 3-dioxygenase 1 ameliorates hippocampal neurogenesis and BOLD-fMRI signals in chronic stress precipitated depression

**DOI:** 10.18632/aging.202511

**Published:** 2021-02-11

**Authors:** Lei Gao, Tingting Gao, Ting Zeng, Peng Huang, Nai-Kei Wong, Zhaoyang Dong, Yunjia Li, Guanghui Deng, Zhiyong Wu, Zhiping Lv

**Affiliations:** 1 School of Traditional Chinese Medicine, Southern Medical University, Guangzhou, Guangdong, China; 2 Foshan Maternal and Child Health Research Institute, Affiliated Hospital of Southern Medical University, Foshan, Guangdong, China; 3 State Key Discipline of Infectious Diseases, Shenzhen Third People’s Hospital, The Second Hospital Affiliated to Southern University of Science and Technology, Shenzhen, Guangdong, China; 4 School of Nursing, Guangzhou University of Chinese Medicine, Guangzhou, Guangdong, China

**Keywords:** depression, IDO1, dorsal raphe nucleus, neurogenesis, blood oxygen level-dependent signal

## Abstract

Indoleamine 2, 3-dioxygenase 1 (IDO1) has been implicated in the pathogenesis of depression, though its molecular mechanism is still poorly understood. We investigated the molecular mechanism of IDO1 in depression by using the chronic unpredictable mild stress (CUMS) model in *Ido1^-/-^* mice and WT mice. The brain blood oxygen level dependent (BOLD) signals in mice were collected by functional magnetic resonance imaging (fMRI) technology. IDO1 inhibitor INCB024360 was intervened in dorsal raphe nucleus (DRN) through stereotactic injection. We found an elevation of serum IDO1 activity and decreased 5-HT in CUMS mice, and the serum IDO1 activity was negatively correlated with 5-HT level. Consistently, IDO1 was increased in hippocampus and DRN regions, accompanied by a reduction of hippocampal BDNF levels in mice with CUMS. Specifically, pharmacological inhibition of IDO1 activity in the DRN alleviated depressive-like behaviour with improving hippocampal BDNF expression and neurogenesis in CUMS mice. Furthermore, ablation of *Ido1* exerted stress resistance and decreased the sensitivity of depression in CUMS mice with the stable BOLD signals, BDNF expression and neurogenesis in hippocampus. Thus, IDO1 hyperactivity played crucial roles in modulating 5-HT metabolism and BDNF function thereby impacting outcomes of hippocampal neurogenesis and BOLD signals in depressive disorder.

## INTRODUCTION

As one of the most prevalent psychiatric disorders worldwide, major depressive disorder (MDD) not only increases the likelihood of negative lifestyle conditions, such as smoking, obesity and drug abuse [1, 2], but also imposes serious mental and economic pressure on the family and society [3]. Virtually all drugs used to treat depression target the same basic mechanisms identified more than 60 years ago, but these existing pharmacotherapies induce full remission in fewer than 50% of patients [4]. Recent neurobiological studies have highlighted the complexity of depression pathogenesis, which may involve dysregulation of the hypothalamic/pituitary/adrenal axis and the monoaminergic system as well as various neurotransmitters/neuromodulators, such as acetylcholine, GABA, substance P, cholecystokinin, and endogenous opioids [5–10]. Despite progress in such mechanistic investigations, the clinical treatment of MDD has remained largely restricted to symptomatic management.

As a central neurotransmitter system, the serotonergic system is responsible for modulating mood and emotion. Dysregulation of this system has been implicated in various psychopathological states including anxiety, depression, impulsivity, and aggression [11, 12]. The essential amino acid tryptophan (TRP) is a common precursor for the biosynthesis of the neurotransmitter serotonin (5-HT) and the metabolite kynurenine (KYN), which play apparently divergent roles in the pathophysiology of MDD [13]. Consistently, hypofunction of the serotonergic system is a salient clinical feature of MDD [14, 15]. It is noteworthy that majority of the 5-HT-producing neurons in the central nervous system (CNS) are located in the dorsal raphe nucleus (DRN) [16]. Histological studies of the DRN in the post-mortem tissues of suicide victims with MDD have revealed altered expression levels of 5-HT-specific markers, such as 5-HT1A and 5-HT2A receptors [17], and of certain transcription factors controlling gene expression in 5-HT neurons [18–21].

Notably, increased activity of indoleamine 2, 3 dioxygenase 1 (IDO1), the first and rate-limiting enzyme of the KYN pathway [22] has been associated with diminished CNS 5-HT content, which is negatively correlated with the severity of depression symptoms [13, 23]. On the other hand, elevated KYN production and neuroplastic changes mediated by its derivatives, such as quinolinic acid, seem to play detrimental roles in the development of depression [24, 25]. These findings raise the possibility that aberrations in TRP metabolism in the DRN (especially in terms of 5-HT content and IDO1 activity) may have strong implications for MDD pathogenesis.

The hippocampus has been extensively studied as a brain region vulnerable to stress and depression [26, 27]. As a pivotal memory processing centre, it is densely innervated by serotonergic neurons of the DRN and is populated by the majority of 5-HT receptor subtypes. Brain-derived neurotrophic factor (BDNF) is ubiquitous in various tissues of the CNS, where it regulates key cellular processes ranging from plasticity to neurogenesis. In MDD, reduced BDNF expression occurs as a consequence of compromised serotonergic signalling, leading to the development of hippocampal dysfunction [28]. Indeed, it has been demonstrated that hippocampal neurons undergo atrophy and downregulation of BDNF expression in response to unmitigated stress [29]. Moreover, our previous studies had demonstrated that depression induced by chronic stress could generate disorder of hippocampal blood oxygen level dependent (BOLD) signals, accompanied by the reduction of BDNF level and the number of adult neurons in the dentate gyrus.

In order to better understand how BDNF downregulation and neurogenesis hypofunction are linked to IDO1 dysregulation in chronically stressed hippocampus, both clinical study and experimental investigation in animal models are warranted. In this study, we conducted WT and IDO1 mutant mice studies with functional magnetic resonance imaging (fMRI) analysis to confirm that IDO1 was the critical regulator in biochemical imbalances of TRP metabolites, and disorder of brain BOLD signals in the pathophysiology of depression. We used the chronic unpredictable mild stress (CUMS) mouse model to reconstitute chronic stress conditions reminiscent of depression in order to testify hypotheses that IDO1 hyperactivity predicts 5-HT reduction, compromised brain function and lowered neurogenesis in the hippocampus in depressive disorder. Our findings thus shed light on a potential strategy for treating depression through pharmacologically targeting an under examined signalling pathway regulating TRP metabolism.

## RESULTS

### IDO1 was increased and accompanied by a reduction of serum 5-HT and hippocampal BDNF levels in CUMS mice

**Figure 1:**
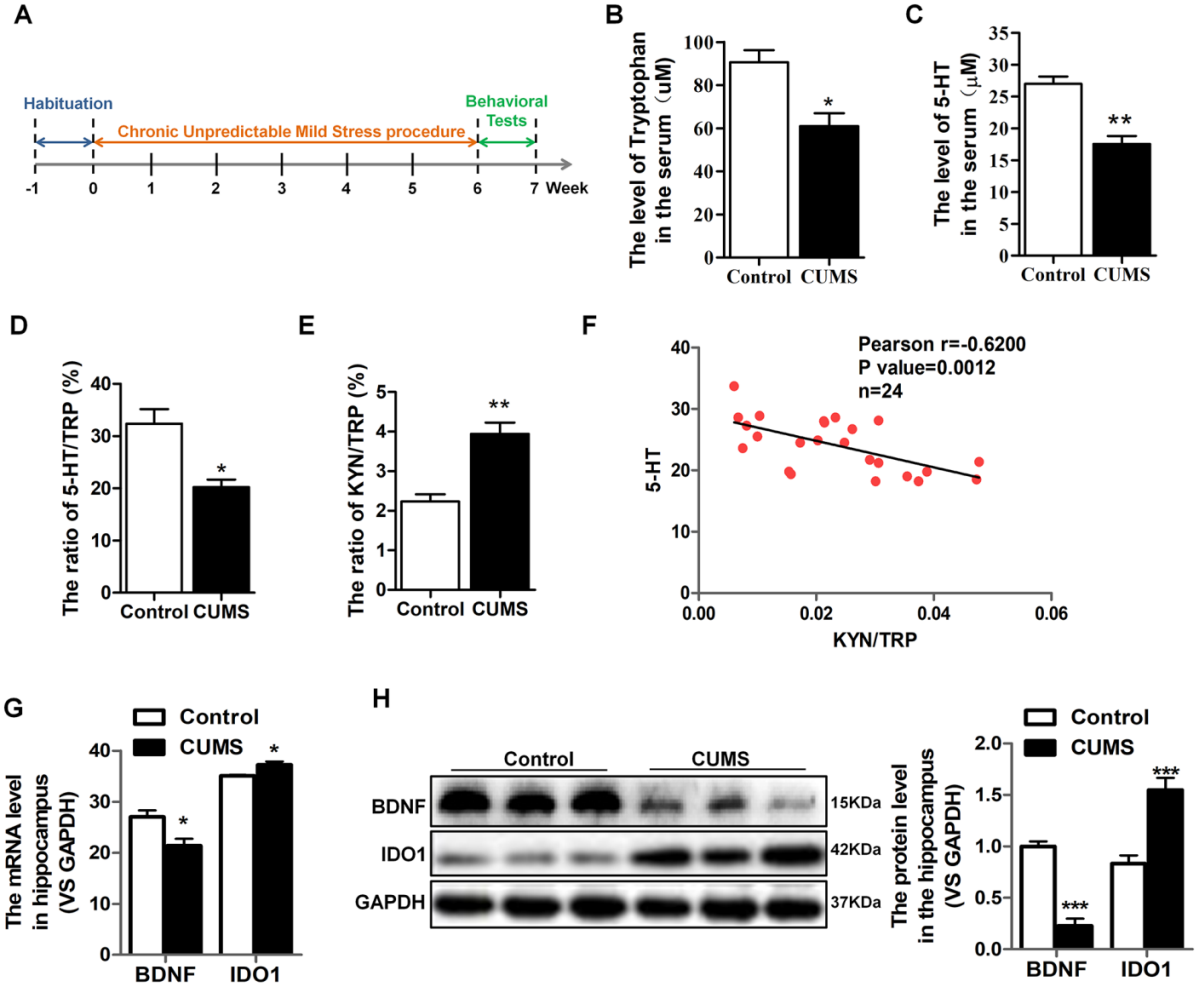
**IDO1 was increased and negatively correlated with serum 5-HT and hippocampal BDNF levels in CUMS mice.** (**A**) Schema for chronic unpredictable mild stress (CUMS) schedule and behavioral experiments. (**B**–**E**) LC-MS detection of the TRP and 5-HT, 5-HT/TRP and KYN/TRP ratio in serum of control and CUMS groups. (**F**) Pearson linear correlation tests for KYN/TRP ratio and 5-HT levels in serum. n=6 mice/group. (**G**) Quantitative real-time PCR analysis of BDNF and IDO1 in the hippocampus. (**H**) Western blot analysis of BDNF and IDO1 in the hippocampus. Bars represent mean±SEM. *p< 0.05, ** *p*< 0.01, *** *p*< 0.001.

An established mouse model of CUMS utilized to characterize TRP metabolism and 5-HT alterations in depression. We carefully collected serum samples from animals with the groups matched in terms of age and gender. The schedule of CUMS induction is as shown in Figure 1A. As expected, the CUMS mice had significant reductions in serum TRP [T=3.572, *P*=0.023] (Figure 1B), 5-HT [T=5.501, *P*=0.005] (Figure 1C) and the 5-HT/TRP ratio [T=3.859, *P*=0.018] (Figure 1D), as well as elevations the KYN/TRP ratio [T=5.093, *P*=0.00469] (Figure 1E) in serum, which was the indicator of the IDO1 activity. Moreover, Pearson’s linear correlation tests revealed that the concentration of serum 5-HT in CUMS mice was negatively correlated with the IDO1 activity (Figure 1F). On the other hand, we also found the IDO1 protein level [T=5.028, *P*=0.001] in the serum of CUMS mouse was significantly elevated compared with control mice (Figure 1H). Remarkably, both qPCR and Western blot analysis reveal a divergent pattern of *BDNF* [T=9.117, *P*<0.001] and *IDO1* in the hippocampus of CUMS mice versus the control group (Figure 1G, 1H); CUMS treatment evidently induced down-regulation of *BDNF* gene [T=3.003, *P*=0.0398] but up-regulation of *IDO1* gene [T=3.127, *P*=0.0353] in the hippocampus.

### IDO1 was up-regulated in DRN region after CUMS administration, accompanied by the reduction of TPH2 levels

**Figure 2:**
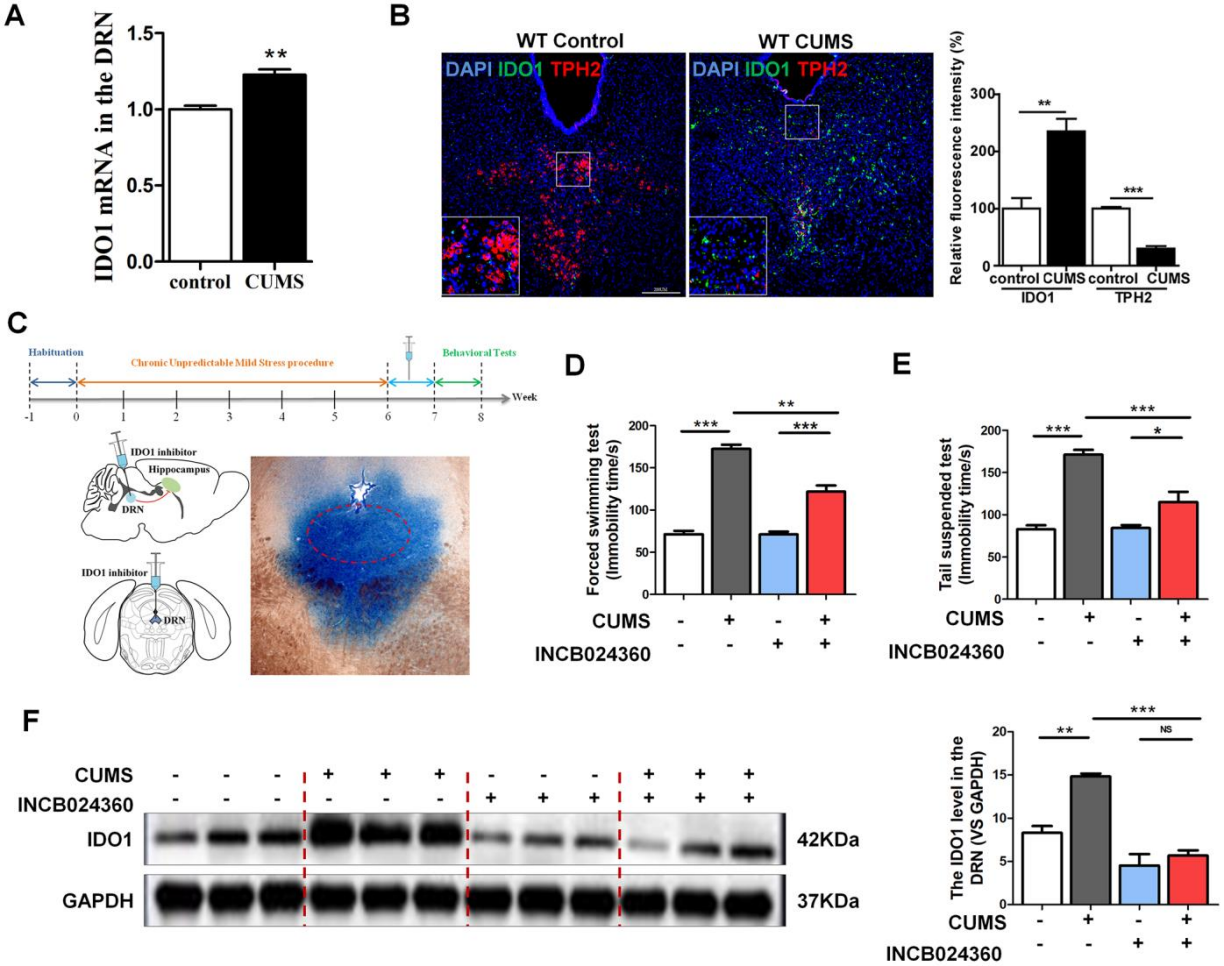
**DRN was a region potentially regulated by IDO1 in depression.** (**A**) Quantitative real-time PCR analysis of IDO1 in the DRN. (**B**) Immunofluorescence analysis of IDO1(green) and TPH2(red) in DRNs of control and CUMS groups (scale bar 200 μm). (**C**) Schema for CUMS schedule, IDO1 inhibitor (INCB024360) injection and behavioral experiments. (**D**) Time immobile in the forced swim assay. (**E**) Time spent immobile in tail suspension assay. (**F**) Western blot analysis of the expressions of IDO1 in the DRN after *in situ* injection with INCB024360. n = 6 mice/group. Bars represent mean±SEM. *p< 0.05, ** *p*< 0.01, *** *p*< 0.001.

As a rate-limiting enzyme in TRP metabolism, IDO1 activity has a negative correlation with 5-HT. It is imperative to note that the majority of the 5-HT-producing neurons in the CNS are located in the DRN [16]. Therefore, we selected the DRN as a target brain region to study the mechanisms through which IDO1 regulates depression. Consistent with previous reports that depression is associated with increased IDO1 expression [30, 31], qPCR analysis showed that CUMS treatment significantly upregulated IDO1 [T=5.237, *P*=0.006] in the DRN (Figure 2A). Tryptophan hydroxylase 2 (TPH2), which is expressed exclusively in neuronal cells, catalyses the initial and rate-limiting steps in the biosynthesis of 5-HT. Given that TPH2 competes with IDO1 for substrates, we next examined the expression of IDO1 and TPH2 in the DRN by immunofluorescence analysis. Consistent with the qPCR result, staining analysis further confirmed that IDO1 expression in the DRN was significantly increased in the CUMS mice compared to that in the control mice (Figure 2B green), while the TPH2 had a significant decrease in the DRN after CUMS exposure (Figure 2B red).

### Pharmacological inhibition of IDO1 in DRN alleviated depressive-like behaviour in CUMS mice

To examine whether inhibition of IDO1 activity would influence depressive behaviours in CUMS-exposed mice, we administered an IDO1-selective inhibitor, INCB024360 (50 mg/d, Selleck, USA) [5], into the DRN after CUMS for 7 consecutive days by standard stereotaxic surgical procedures (Figure 2C). For analysis, we set up 4 groups: control, CUMS, control+INCB024360 and CUMS+INCB024360. Multiple lines of evidence from our study suggest that inhibition of IDO1 activity in the DRN attenuates depressive-like behaviour. As shown in Figure 2D, one-way ANOVA showed significant differences in FST [F=88.936*P*<0.001] immobility time among the groups. CUMS exposure significantly increased the FST immobility time compared with that of the control animals [*P*<0.001]. However, treatment with CUMS+INCB024360 induced a decrease in immobility time compared to that of the mice exposed to CUMS alone [*P*<0.001]. In addition, in the TST [F=31.633, *P*<0.001], the immobility time of the mice in the CUMS group was remarkably increased compared with that in the control group [*P*<0.001], while a decrease was shown in the CUMS+INCB024360 group compared with the CUMS group [*P*=0.003] (Figure 2E). Furthermore, western blot analysis showed that the inhibition of IDO1 activity in the DRN increased the IDO1 protein level [F=30.117, *P*<0.001] in the DRN (Figure 2F).

### Inhibition of IDO1 activity in the DRN improved the BDNF expression and neurogenesis in the dentate gyrus of hippocampus after CUMS treatment

**Figure 3:**
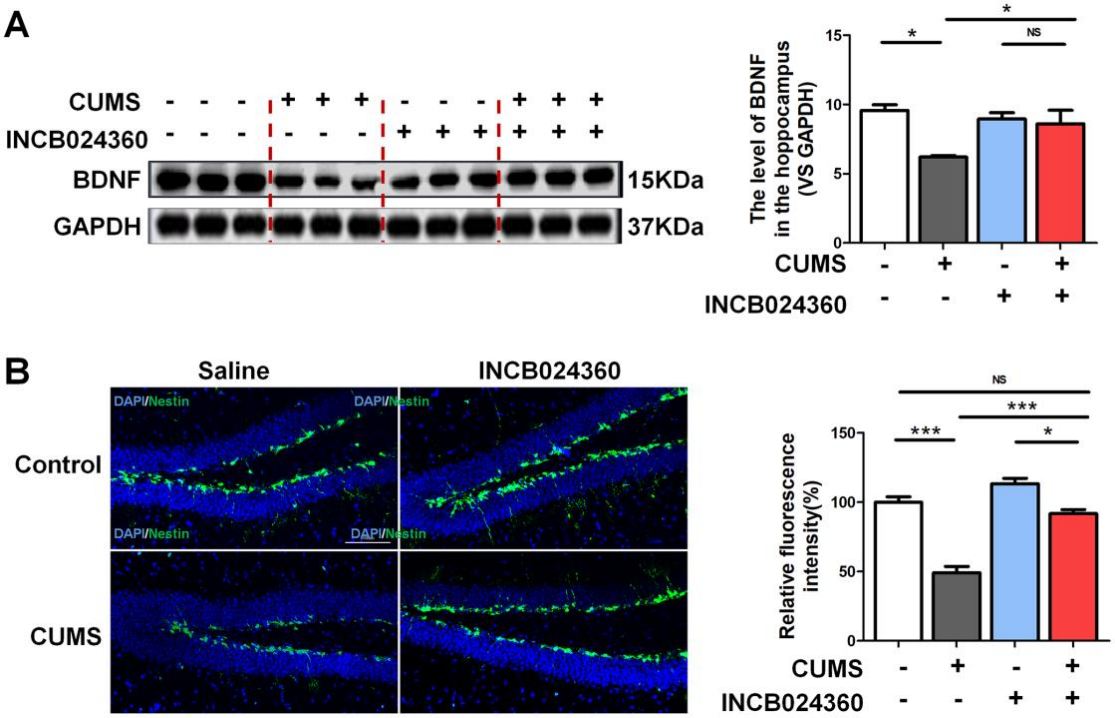
**Inhibition of IDO1 activity in the DRN improved the BDNF expression and neurogenesis in the dentate gyrus of hippocampus after CUMS treatment.** (**A**) Western blot analysis of BDNF in the hippocampus of Control, CUMS, Control + INCB024360 and CUMS + INCB024360 groups. (**B**) IF analysis of the numbers of Nestin-positive stem cell in the dentate gyrus of hippocampus after INCB024360 treatment (scale bar 100 μm). n = 6 mice/group. Bars represent mean±SEM. *p< 0.05, ** *p*< 0.01, *** *p*< 0.001.

Surprisingly, the western blot analyses showed that the level of BDNF [F=6.481, *P*=0.016] in the hippocampus was increased in the CUMS+INCB024360 group compared with that in the CUMS-only group [P=0.366] (Figure 3A). To assess the effect of the INCB024360 treatment on the survival of neuronal cells in the hippocampus of the CUMS-exposed animals, we next determined the number of Nestin-positive stem cell [F=50.096 *P*<0.001] in the dentate gyrus of hippocampus on day 7 post-INCB024360 treatment. The CUMS animals showed a decrease in the number of Nestin-positive in the dentate gyrus (Figure 3B). As expected, INCB024360 was effective at increasing the number of Nestin-positive in the dentate gyrus, suggesting that IDO1 inhibition was effective for promoting hippocampal neurogenesis under chronic stress.

### IDO1 genetic ablation reduced the depression-like phenotypes in mice under CUMS treatment

**Figure 4:**
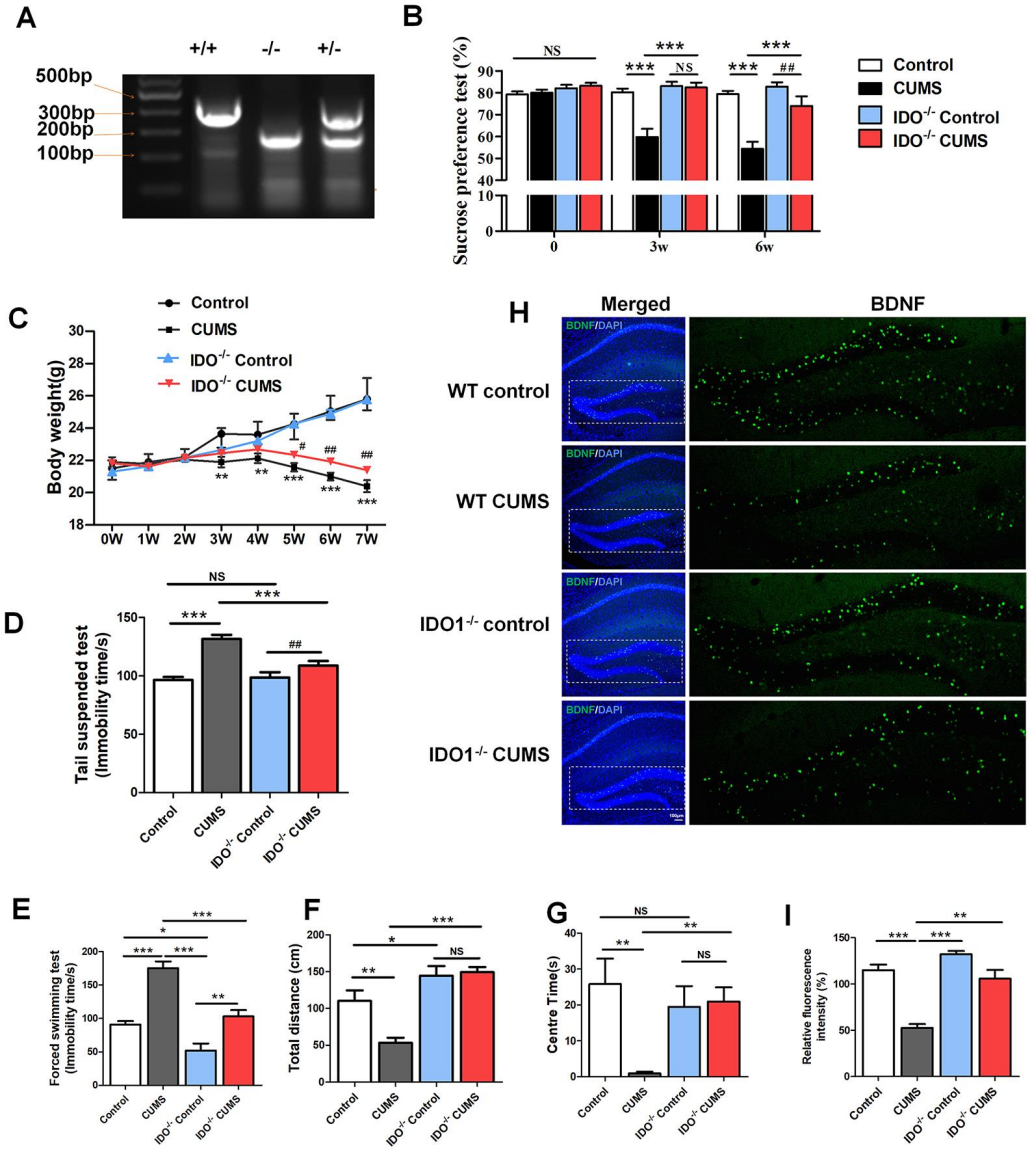
**IDO1 genetic ablation reduced the depression-like phenotypes in mice under CUMS treatment.** (**A**) Gene identification. (**B**) The body weight change of mice during CUMS procedure, *p< 0.05 vs. WT control group; ^#^ *p* < 0.05 vs. WT CUMS group. The behavior analysis by (**C**) the sucrose consumption test, (**D**) tail suspension assay, (**E**) forced swim assay and (**F**, **G**) open field test in WT and *Ido1*^-/-^ mice. (**H**, **I**) Immunofluorescence analysis of the BDNF protein (green) expression in the hippocampus of WT and *Ido1*^-/-^groups. Bars represent mean±SEM. *p< 0.05, ** *p*< 0.01, *** *p*< 0.001.

Considering that highly expressed IDO1 has been associated with depression, we next used *Ido1*^-/-^ mice with or without CUMS administration to assess body weight and behavioural changes and TRP metabolism. Confirmation of IDO1 gene knockout was shown in Figure 4A. In terms of sugar preference, the *Ido1*^-/-^ mice with 3w CUMS administration had normal glucose preferences [F=18.681, *P*<0.001], unlike the WT CUMS mice (Figure 4B). Even though after 6w CUMS treatment [F=18.93 1, *P*<0.001], the *Ido1*^-/-^ mice also appeared a decrease of glucose preferences, it was significantly higher than WT CUMS mice (Figure 4B). The body weight of different group mice was recorded for 7 weeks. The CUMS treatment had induced significant decrease of the body weight both in WT and *Ido1*^-/-^ mice (Figure 4C). However, the *Ido1*^-/-^ CUMS mice had a more stable body condition with less weight loss than WT CUMS mice [*P*=0.04] during the later period of the CUMS procedure (Figure 4C). In the tail suspension test (TST) [F=12.804, *P*<0.001], the immobility time of the *Ido1*^-/-^ group after CUMS administration was significantly reduced [*P*=0.002] compared with that of WT CUMS group (Figure 4D). In the forced swim test (FST) [F=30.966, *P*<0.001], the CUMS mice exhibited immobility time results similar to their TST results (Figure 4E). Interestingly, the *Ido1*^-/-^ mice were more active in the FST than the WT mice [*P*=0.002], and their immobility time was shortened compared with that of the control and CUMS group (Figure 4E). In addition, the open field test also indicated that *Ido1*^-/-^ mice were more activity and boldness than the WT mice with the longer distance moved [F=18.077, *P*<0.001] and more time in central arena [F=3.643, *P*=0.022] after the CUMS administration (Figure 4F, 4G). Moreover, we found that CUMS-exposed *Ido1*^-/-^ mice had not significant decrease of BDNF protein in hippocampus compared to control mice, while a reduction in BDNF was seen in WT CUMS mice based on the immunostaining analysis (Figure 4H, 4I). These results indicated that the existent of *Ido1* gene was essential for the depressive-like behaviour and biochemical phenotypes in mice with the CUMS exposure.

### Deletion of the IDO1 retrieves hippocampal BOLD signal in mice under CUMS treatment

**Figure 5:**
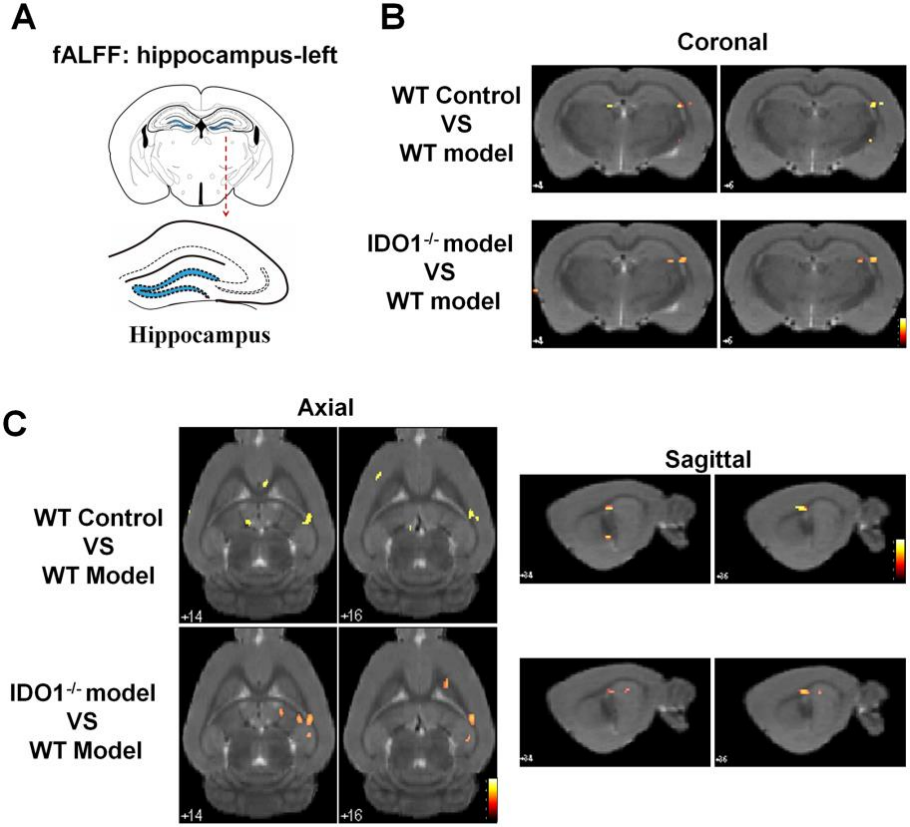
**Deletion of the IDO1 retrieves hippocampal BOLD signal in mice under CUMS treatment.** (**A**) Sketch of the hippocampus. (**B**, **C**) Images of coronal, axial and sagittal planes show the changes of BOLD–fMRI signal in the hippocampal region between different groups according to the fALFF analysis. The voxel-level height threshold was *p < 0.005* (uncorrected) and the cluster-extent threshold was 20 voxels.

Based on our previous studies, the hippocampus was the key region with the dysfunction of BOLD signals in depressive mice. As shown in Figure 5, according to the fALFF analysis, the hippocampal BOLD signals were significant activation in control mice under the coronal (Figure 5B), axial (Figure 5C, left) and sagittal planes (Figure 5C, right) compared with the CUMS group. Interestingly, even though after CUMS administration, IDO1 KO mice still shown a similar hippocampal BOLD signals condition to the control mice with the almost same area in coronal, axial and sagittal planes (Figure 5B, 5C, lower). These results indicated that the loss of IDO1 prevented the CUMS induced disorders of bold signal in hippocampus.

### IDO1 genetic ablation ameliorates neurogenesis in mice under CUMS treatment

**Figure 6:**
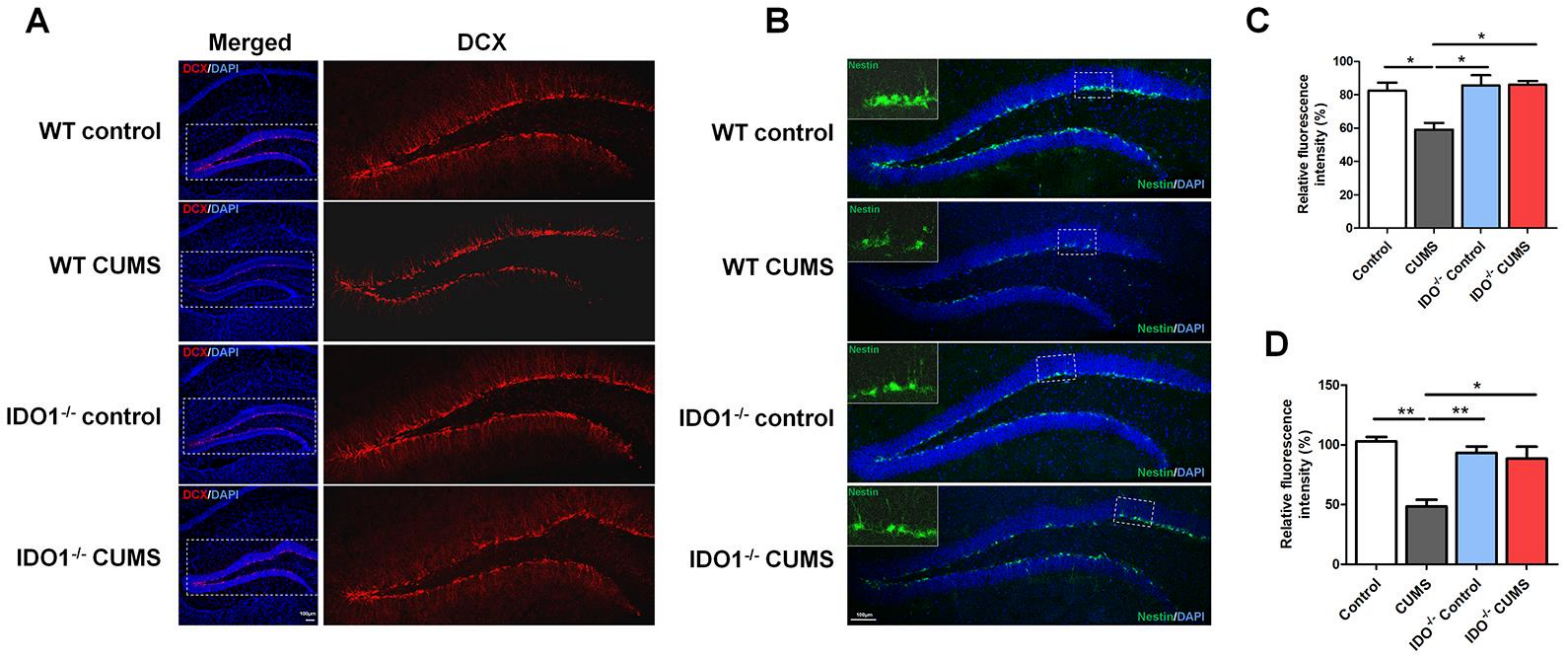
**IDO1 genetic ablation ameliorates neurogenesis in mice under CUMS treatment.** (**A**, **C**) Immunostaining and the relative fluorescence intensity of the DCX in the hippocampus of WT and *Ido1*^-/-^groups. (**B**, **D**) Immunostaining and the relative fluorescence intensity of the Nestin in the hippocampus of WT and *Ido1*^-/-^groups. (scale bar 100 μm). n = 6 mice/group. Bars represent mean±SEM. * *p*< 0.05, **p< 0.01.

To validate the effect of the IDO1 genetic manipulation on the neurogenesis in hippocampus with the CUMS-exposure, histological study by IF staining was performed in WT and *Ido1*^-/-^ groups to visualize the proteins of interest. We further detected immature neurons with specific markers (Nestin and DCX) in the dentate gyrus of hippocampus. As expected, micrographs of hippocampus slices showed that the number of DCX-positive neurons [F=7.799, *P*=0.009] was up-regulated in the dentate gyrus of *Ido1*^-/-^ CUMS mice compared to the WT CUMS group (Figure 6A, 6C), as well as the Nestin-positive [F=13.58, *P*=0.002] cells (Figure 6B, 6D). These results indicated that IDO1 deficiency had the potential role against depression by improving the neurogenesis with the immature neurons in hippocampus. Taken as a whole, these findings corroborate the notion that the *Ido1*^-/-^ mice had the ability of resisting to the CUMS induced depression associated with hippocampus function and neurogenesis, suggesting that IDO1 may be a powerful target for controlling depression-related phenotypes.

## DISCUSSION

We have demonstrated that IDO1 expression is regulated in a pattern virtually opposing that of BDNF expression in the hippocampus and DRN in mice displaying depressive-like behaviors. As expected, CUMS administration induced an elevation of IDO1 activity, indicated by KYN/TRP ratio, was accompanied by a reduction of 5-HT levels in the serum of depressive mice. These results supported a plausible relationship between IDO1 hyperactivity and a model of chronic stress induced depression. Moreover, BDNF down-regulation was accompanied by IDO1 up-regulation in the hippocampus and DRN regions in depressed mice with CUMS administration. We demonstrated that DRN is potentially the region regulated by IDO1 in 5-HT metabolism and hippocampal neurogenesis in mice with depression-like phenotypes. Our study provides direct evidence that genetic ablation or pharmacological inhibition of IDO1 were sufficient to attenuate KYN-related neurotoxicity *in vivo* and raise the threshold for developing depressive behaviors, led to an increase in neurogenesis and amelioration of behavioral deficits, in conjunction with a rebound in expression level of BDNF in the hippocampus. Specifically, the aberrant BOLD-fMRI signals in hippocampus induced by the CUMS exposure were also rectified by absence of IDO1. These results indicated that IDO1 hyperactivity played crucial roles in modulating TRP/5-HT metabolism and BDNF function thereby impacting outcomes of hippocampal neurogenesis and BOLD signals in depressive disorder.

Two major TRP metabolites produced via enzymatic regulation, 5-HT and KYN, have been implicated in the mechanisms of depression [32, 33]. Recent report confirms that KYN and the KYN/TRP ratio are strongly related to neuropsychological performance in depression [34]. Consistent with findings from clinical studies, the LC-MS/MS results of animal serum suggest that the CUMS mice had significant reductions in TRP, 5-HT and the 5-HT/TRP ratio and elevations in the KYN/TRP ratio. A high KYN/TRP ratio indicated IDO1 hyperactivity. Moreover, IDO1 was negatively correlated with serum 5-HT and hippocampal BDNF levels in CUMS mice. Therefore, we raise the possibility that potential therapeutic intervention for depression might be possible through targeting IDO1 activity, as an alternative to the current approach of symptomatic management with antidepressants.

Epidemiological studies have supported a strong functional link between BDNF and 5-HT in MDD(36), with 5-HT being a pivotal neurotransmitter regulating BDNF function in the hippocampus. The present study supports the notion that IDO1 antagonizes BDNF effects at the behavioral level in depression. Considering that negative correlation between 5-HT and IDO1 has been associated with depression, it is imperative to note that the majority of the 5-HT-producing neurons in the CNS are located in the DRN [16, 35]. Studies have suggested that the DRN projects to hippocampus and the severity of depressive symptoms correlates with DRN-amygdala/hippocampus connectivity [36, 37]. Therefore, we hypothesize that DRN is potentially a brain area where IDO1 regulates depression. As expected, qPCR and western blot analyses indicated that IDO1 expression in DRN tissue was increased in the CUMS mice compared to that in the control mice. IF analysis further verified that the CUMS animals had an obvious decrease in TPH2 and increase in IDO1 in the DRN. These data provide evidence supporting our hypothesis. To examine whether inhibition of IDO1 activity would influence depressive behaviours in CUMS-exposed mice, we further confirmed that inhibition of IDO1 activity in the DRN attenuated depressive-like behaviour and prevented down-regulation of BDNF and neurogenesis in hippocampus after CUMS treatment. The current findings from animal experiments suggest a novel mechanistic link between IDO1 in the DRN and depression, which the detrimental effects of IDO1 hyperactivity are likely mediated through a loss of hippocampal BDNF expression.

Epidemiological studies have supported a strong functional link between BDNF and 5-HT in MDD, with 5-HT being a pivotal neurotransmitter regulating BDNF function in the hippocampus [38]. As noted, gene knockout or pharmacological inhibition of IDO1 promoted stable normal expression of BDNF and immature neurons in the hippocampus. Our experimental results shown that deletion of IDO1 could effectively improve depression behaviour in the CUMS mice and indicated that IDO1 ablation could be a powerful method for controlling depression-related phenotypes. Surprisingly, based on fMRI analysis, we also found that loss of IDO1 prevented the CUMS induced disorders of BOLD signals in hippocampus, suggesting that hippocampus might be the specific region regulated by IDO1 in depression. Therefore, as a key TRP metabolism enzyme, IDO1 plays a critical role in the pathogenic mechanisms of depression associated with the regulation of endogenous KYN and 5-HT biosynthesis. Downregulation of 5-HT signalling in the hippocampus can be logically anticipated as a result of IDO1 hyperactivity in the DRN, leading to compromised 5-HT-dependent expression of BDNF in the hippocampus. These results are consistent with these findings and further indicate that a high KYN/TRP ratio, as a function of IDO1, predicts reduced BDNF levels and aberrant BOLD signals in the hippocampus.

Our findings highlight an antidepressant effect by injection of IDO1 inhibitor into the DRN of mice and raise the possibility that IDO1 is involved in controlling depression in humans and may thus represent a novel drug target. This new strategy is based on the prevention or reversal of depression-like phenotypes by targeting IDO1, whose activity and expression are pathophysiologically linked to imbalances in TRP metabolites in the brain. Since there are different subtypes of depression, future investigation is warranted to test the more detail mechanisms of IDO1 in brain functional regulation and preclinical settings with human subjects and to validate the robustness of IDO1 intervention as a therapeutic strategy.

## MATERIALS AND METHODS

### Animals

Adult C57BL/6J mice and *Ido1*^-/-^ mice (strain IDO1^tm1Alm^/J, Jax Strain #005867) were bred at the Animal Experimental Centre of Southern Medical University. All animals were maintained in a temperature- (21 ± 2° C) and humidity (55% ± 5%)-controlled room with a 12-h light-dark cycle, with food and water provided *ad libitum*. Eight-week-old mice were used at the start of the experiments. All animal studies were performed under the approval of the National Institutional Animal Care and Ethics Committee of Southern Medical University.

### CUMS procedure

The CUMS procedure was conducted as previously reported [39, 40]. The procedure was based solely on environmental and social stressors randomly arranged day and night across 42 consecutive days. The CUMS procedure was performed as described in Supplementary Material.

### Behavioural testing, fMRI data acquisition and analysis

Tests of sucrose preference and fear conditioning, as well as the fMRI data acquisition were conducted following the previous reports [30, 31] and described in the Supplementary Material.

### RNA and protein analysis

Quantitative real-time polymerase chain reaction (qPCR) and western blotting were performed as previously reported [41]. Immunofluorescence was performed on free-floating sections and confocal images were acquired by laser scanning confocal microscope (C2+, Nikon, Japan). The primers and antibody used were shown in the Supplementary Tables 1, 2.

### Drug administration

IDO1 inhibitor (INCB024360, 1mg/(kg.d)) was continuously injected into the DRN for 7 consecutive days after CUMS with the aid of a microinjection pump (RWD Life Science, China).

### Stereotactic injection

Methods used in this study were based on modified protocols [42–44] for stereotactic injection into the DRN of mice. Mice were anaesthetized by a combination of ketamine (0.1 mL/100 g, 100 mg/kg), xylazine (0.01 mL/100 g, 2 mg/kg) and midazolam (0.05 mL/100 g, 0.5 mg/kg). Anaesthetic doses appropriate to the mouse’s body weight (0.1 mL/10 g) were administered intraperitoneally. Then, the animal was placed in a stereotaxic instrument (RWD Life Science, China). Erythromycin eye ointment was applied to prevent corneal drying, and a heating pad (RWD, China) was used to maintain the body temperature at 37° C. A small craniotomy hole was made of a dental drill (OmniDrill35, WPI). A micropipette (KDS310, USA) connected to a Quintessential Stereotaxic Injector was used for injection. IDO1 inhibitor (INCB024360, 1mg/(kg.d)) was injected into the DRN (2 μL per injection; AP: 5.2 mm; ML: ± 0 mm; DV: 2.7 mm, with a 15° angle) for 7 consecutive days after CUMS, while WT control mice were injected with 0.9% NaCl solution. Following injection, the wound was sutured. Antibiotics (bacitracin and neomycin) were applied to the surgical wound, and ketoprofen (5 mg/kg) was injected subcutaneously. The animals were then allowed to recover from anaesthesia under a heat lamp.

### LC-MS/MS

Analysis by LC–MS/MS was conducted as detailed in the Supplementary Material.

### Statistical analysis

Data were analysed with the SPSS software (version 20.0) and were presented as Mean ± SEM. Statistical analysis was performed by using an unpaired *t*-test or one-way ANOVA followed by Tukey’s multiple-comparisons test depending on experimental designs. Correlation tests were performed via Pearson's coefficient test for linear regression. Statistically significance was determined at *p*< 0.05.

### Ethical standards

The authors assert that all procedures contributing to this work comply with the ethical standards of the relevant national and institutional guides on the care and use of laboratory animals.

## Supplementary Material

Supplementary Materials and Methods

Supplementary Tables
