# Supplementary material for: Blockade of Indoleamine 2, 3-dioxygenase 1 ameliorates hippocampal neurogenesis and BOLD-fMRI signals in chronic stress precipitated depression
Source: Aging (Albany NY). 2021 Feb 11;13(4):5875–91. doi: 10.18632/aging.202511 (PMC7950278; doi:10.18632/aging.202511)
Supplement: Supplementary Materials and Methods [file aging-13-202511-s001.pdf]

SUPPLEMENTARY MATERIALS AND METHODS

Animals

Adult male C57BL/6J mice and *Ido*$^{-/-}$ mice (strain IDO1^{tm1Alm}/J, Jax Strain #005867) were bred at the Animal Experimental Center of Southern Medical University. All animals were bred in a temperature (21 ± 2° C) and humidity (55% ± 5%) controlled room with a 12-h light dark cycle, with food and water provided *ad libitum*. Mice were assigned into four groups (*n*=12-20 per group): (1) WT control group; (2) WT CUMS group, which was imitating adult stress; (3) *Ido*$^{-/-}$ control group and (4) *Ido*$^{-/-}$ CUMS group. The body weight, food and water consumption of each mouse were recorded weekly. All animal studies were performed under the approval of the National Institutional Animal Care and Ethical Committee of Southern Medical University.

Chronic unpredictable mild stress (CUMS) procedure contained 9 different stressors randomly arranged day and night across 48 consecutive days: (1) 24 hours food and water deprivation, (2) 1 hour empty bottle, (3) 17 hours of 45° cage tilt, (4) overnight illumination, (5) 24 hours wet cage, (6) 5 min swimming in water at 4° C, (7) 24 hours disrupting the squirrel cage, (8) 24 hours foreign body stimulation, (9) 4 hours restrict movement. The behavioral tests were performed and scored by trained and experienced observers who were blind to the condition of the animals.

Behavioral tests

The behavioral tests were performed and scored by trained and experienced observers who were blind to the experimental conditions of the animals. At the end of the 6th week, all animals were subjected to behavioral tests. The Sucrose preference test (SPT) was administered 3 times: before stress (baseline), in the middle of the experiment (week 3), and at the end of the experiment (week 6). The Tail Suspension Test (TST) [1], Forced Swim Test (FST) [2] and Open field test (OFT) were carried out after final SPT. The behavioral tests were performed and scored by 3 trained and experienced observers.

Sucrose preference test

Mice were habituated to drink water from two 50 ml falcon tubes with sipper tops for 2 consecutive days. On the third day, mice were given a free choice between two bottles containing either 1 % sucrose in water or regular tap water. The consumed volume of each solution was measured daily during 4 consecutive days and the position of the bottles was interchanged after each daily measurement. Sucrose preference was estimated by dividing the volume of consumed sucrose by the volume of consumed sucrose + consumed tap water. The average of sucrose preference of the 4 test days was calculated to estimate the total preference for sucrose. Mice that did not habituate to drink from the water bottle were not included in this study.

Tail suspension test

The tail suspension test was used to assay depression behavior in mice. Mice were suspended upside-down from their tail for 6 min. The session was recorded by a video camera and the total time immobile was scored.

Forced swim test

Experiments were performed in a glass cylinder (14 cm inner diameter, with water level 13 cm deep) filled with room temperature water (22-23° C). The legs of the mice were unable to touch the bottom of the cylinder. Mice were gently placed into the water and swimming behaviors were video-recorded for 6 min. The total duration of immobility was scored during the last 4 min. Immobility was defined as no movement of the front or back legs and no attempt to escape.

Open field test

Locomotor activity was measured for each animal by open field test. The mice (n = 10/group) were submitted to open field apparatus, consisted of a 40 cm × 40 cm gray box with 40 cm high boundary walls. Each animal was placed softly into the apparatus and observed for 5 min. After each trial, the apparatus was cleaned with 75% ethanol. The central time (an area of 20cmx20cm right in the centre of box) was also traced and recorded by Smart 3.0 (Panlab Spain).

Quantitative real-time PCR (qPCR) analysis of *BDNF* and *IDO1* mRNA

The total RNA was extracted using Trizol reagent (Sigma Aldrich, MO, USA). cDNA was synthesized using a High Capacity RNA-to-cDNA kit (TAKARA, JP) according to the manufacturer's protocol. Each brain sample was analyzed in three independent experiments for each gene. The values obtained for the *BDNF* mRNA and *IDO1* mRNA expression were normalized to GAPDH and quantified relative to the

expression in the control samples. The products were analyzed with densitometry using the Multiplex Quantitative PCR System (MX3005P™, Stratagene, USA).

Western blotting analysis of mice hippocampus tissue

Frozen hippocampus tissues were homogenized in ice-cold RIPA buffer containing 1% protein inhibitors and 1% phosphatase inhibitors. The dissolved proteins were collected after centrifugation at 10,000 × g for 10 min at 4° C, and the supernatant was then collected. Protein concentrations were determined using a BCA Protein Assay Kit (Thermo). Equal amounts of protein were isolated by 10% SDS-PAGE and transferred onto a nitrocellulose membrane. The membrane was blocked with 5% fetal bovine serum albumin in a washing buffer (Tris-buffered saline containing 0.1% v/v Tween-20) for 2 h at 25° C, and subsequently incubated overnight with the primary antibodies against GAPDH (1:2000, Millipore, Massachusetts, USA), BDNF (1:1000, Abcam, UK) and IDO1 (1:1000, Millipore, Massachusetts, USA) were employed. Each membrane was thrice rinsed for 5-10 min each and incubated with the secondary horseradish peroxidase-linked antibodies. Bands corresponding to proteins of interest were scanned and band density was analyzed by using the Quantity One automatic imaging analysis system (Bio-Rad Laboratories, Hercules, CA, USA).

Mice brain tissue immunofluorescence

Mice were first perfused with 50 mL of room-temperature PBS (pH 7.4), and fixed with 75 mL of 4% PFA at 4° C (TAAB, pH 7.3; 25 mL at ~10 mL/min and 50 mL at ~ 6 mL/min). After 8h post-fixation, 40 micrometer-thick coronal sections were cut and kept in PBS 0.1 until use. All reagents were diluted in PBS 0.1 M containing triton X-100(0.2% v/v). Free-floating sections were blocked in 20% normal goat serum (NGS, Vector laboratories) for 1 h at room temperature and incubated at 4° C, and then incubated with the primary antibody against BDNF(1:200, Abcam, UK), IDO1(1:100, Millipore, USA), TPH2 (1:200, CST, USA)and NeuN (1:400, CST, USA)at 4° C overnight. Sections were washed and incubated at room temperature for 1h in 1:500 Alexa 633-conjugated goat anti-rabbit and 1:250 Alexa 488-conjugated anti-GFP rabbit antibody (Invitrogen) with 2% NGS. After extensive washes, sections were mounted in Vector shield (Vector Laboratories). Confocal images were acquired (Leica DM2500 TCS SPE 40x immersion 1.3 NA objective) for each animal, from different sections. Sequential data acquisition was used to prevent cross-talk between Alexa 488 and Alexa 633 signals.

LC-MS/MS

TRP, 5-HT, KYN, QUIN, KYNA, and DHBA as well as formic acid (FA), trifluoroacetic acid (TFA) and methanol (MeOH; FLUKA, LC–MS grade) were obtained from Sigma Aldrich (Steinheim, Germany). The LC–MS/MS system consisted of a CTC HTC PAL Autosampler (CTC Analytics AG, Zwingen, Switzerland) and an Agilent 1200 Series LC system (Micro Vacuum Degasser, Binary Pump SL, Thermostatted Column Compartment; Agilent Technologies, Waldbronn, Germany), coupled to an API 4000TM triple quadrupole mass spectrometer (AB Sciex, Darmstadt, Germany). Data acquisition and processing were carried out by using Analyst® software Version 1.6.2.Stock solutions were prepared individually for each standard and stock solutions in a final concentration of 1 mmol/L. Then, the KYN was diluted to 100 g/mL, QUIN and KYNA diluted to 10 g/mL with pure water as standard solutions, and TRP and 5-HTdirectly with 1mg/mL as a working fluid. All standard stocks were prepared on ice. Aliquots were aerated with nitrogen and stored at -80° C until use. 5 µL standard and serum were put into the chromatographic column, followed by recording of the chromatograms of TRP, KYN, 5-HT, and QUIN, and calculation of their concentrations.

Stereotactic injection

We consulted several articles [3–5] and improved the technique of Stereotactic injection in DRN of mice. Mice were anesthetized by a combination of ketamine (0.1 ml/100 g, 100mg/kg), xylazine (0.01 ml/100g, 2 mg/kg) and midazolame (0.05 ml/100 g, 0.5 mg/kg), prepared under sterile conditions with 0.9% sodium chloride. Appropriate to the body weight (0.1 ml/10 g) anesthetic was administered intraperitoneally. Then, the animal was placed in a stereotaxic instrument (RWD Life Science, Shenzhen, China). Erythromycin eye ointment was applied to prevent corneal drying and a heat pad (RWD, Shenzhen, China) was used to keep body temperature at 37° C. A small craniotomy hole was made by using a dental drill (OmniDrill35, WPI) and a micropipette (KDS310, USA) connected to a Quintessential Stereotaxic Injector for injection. Then, IDO1 inhibitor (INCB024360, 50mg/d) was injected into the DRN (0.2 ml per injection; AP: 5.2 mm; ML: ±0 mm; DV: 2.7 mm, with a 15 angle) for 7 consecutive days after CUMS, while WT control mice were injected with 0.9% NaCl solution. Following injection, the wound was sutured and antibiotics (bacitracin and neomycin) were applied to the surgical wound and ketoprofen (5 mg/kg) was injected subcutaneously. Animals were then allowed to recover from anesthesia under a heat lamp.

fMRI data acquisition and analysis

fMRI scan was performed after behavioural tests as previous studies [6, 7]. Mice were anaesthetized with isoflurane and fixed in a custom-made MRI-compatible cradle with ear and bite bars to minimize head motion. The mice body temperature and respiration rates were measured using a pressure-sensor (SAII Instruments, Model 1030 Monitoring and Gating System, USA). fMRI data were acquired by a 7.0-T animal MRI scanner (70/16 PharmaScan, Bruker Biospin GmbH, Germany) as previous studies, which used a 38-mm birdcage rodent brain quadrature resonator for radiofrequency transmission and reception. An echo-planar imaging EPI sequence with the following parameters was used: protocol = ax-T1w, resolution = 0.14 mm × 0.14 mm × 1.0 mm, matrix size = 192 × 128, slice thickness = 1.40 mm, slice gap = 0.10 mm, repetition time = 603.94 ms, echo time = 9.01 ms, averages = 32, scan time = 5 m 10 s, repetitions = 1, and volume = 1. Image were pre-processed and statistically analyzed using spmratIHEP based on the statistical parametric mapping (SPM12) (http://www.fil.ion.ucl.ac.uk/spm) software and DPARSF (http://rfmri.org/DPARSF) software. The fALFF measures were analyzed and compared between the WT and $Ido^{-/-}$ groups. All the functional images post-processing was performed by a single experienced observer, unaware to whom the scans belonged. The voxel-level height threshold was $P < 0.005$ (uncorrected), and the cluster-extent threshold was 20 voxels.

REFERENCES

1. Yau SY, Li A, Hoo RL, Ching YP, Christie BR, Lee TM, Xu A, So KF. Physical exercise-induced hippocampal neurogenesis and antidepressant effects are mediated by the adipocyte hormone adiponectin. Proc Natl Acad Sci USA. 2014; 111:15810–15. https://doi.org/10.1073/pnas.1415219111 PMID:25331877

2. Russo E, Citraro R, Davoli A, Gallelli L, Di Paola ED, De Sarro G. Ameliorating effects of aripiprazole on cognitive functions and depressive-like behavior in a genetic rat model of absence epilepsy and mild-depression comorbidity. Neuropharmacology. 2013; 64:371–79. https://doi.org/10.1016/j.neuropharm.2012.06.039 PMID:22766393

3. Tejedor LS, Wostradowski T, Gingele S, Skripuletz T, Gudi V, Stangel M. The effect of stereotactic injections on demyelination and remyelination: a study in the cuprizone model. J Mol Neurosci. 2017; 61:479–88. https://doi.org/10.1007/s12031-017-0888-y PMID:28124770

4. McSweeney C, Mao Y. Applying stereotactic injection technique to study genetic effects on animal behaviors. J Vis Exp. 2015; e52653. https://doi.org/10.3791/52653 PMID:25992973

5. Suryawanshi H, Sarangdhar MA, Vij M, Roshan R, Singh VP, Ganguli M, Pillai B. A simple alternative to stereotactic injection for brain specific knockdown of miRNA. J Vis Exp. 2015; e53307. https://doi.org/10.3791/53307 PMID:26779762

6. Huang P, Dong Z, Huang W, Zhou C, Zhong W, Hu P, Wen G, Sun X, Hua H, Cao H, Gao L, Lv Z. Voluntary wheel running ameliorates depression-like behaviors and brain blood oxygen level-dependent signals in chronic unpredictable mild stress mice. Behav Brain Res. 2017; 330:17–24. https://doi.org/10.1016/j.bbr.2017.05.032 PMID:28527694

7. Gao L, Huang P, Dong Z, Gao T, Huang S, Zhou C, Lai Y, Deng G, Liu B, Wen G, Lv Z. Modified Xiaoyaosan (MXYS) Exerts Anti-depressive Effects by Rectifying the Brain Blood Oxygen Level-Dependent fMRI Signals and Improving Hippocampal Neurogenesis in Mice. Front Pharmacol. 2018; 9:1098. https://doi.org/10.3389/fphar.2018.01098 PMID:30323763
