# Supplementary material for: Blockade of Indoleamine 2, 3-dioxygenase 1 ameliorates hippocampal neurogenesis and BOLD-fMRI signals in chronic stress precipitated depression
Source: Aging (Albany NY). 2021 Feb 11;13(4):5875–91. doi: 10.18632/aging.202511 (PMC7950278; doi:10.18632/aging.202511)
Supplement: Supplementary Tables [file aging-13-202511-s002.pdf]

# SUPPLEMENTARY TABLES

**Supplementary Table 1. Sequences and annealing temperatures of the oligo primers used in this study.**

| | Primer sequence | | Annealing temperature |
|---|---|---|---|
| | Forward (5’->3’) | Reverse (5’->3’) | |
| IDO1 | CAAAGCAATCCCCACTGTATCC | ACAAAGTCACGCATCCTCTTAAA | 60° C |
| GAPDH | AAGGGCTCATGACCACAGTC | GGATGCAGGGATGATGTTCT | 60° C |

**Supplementary Table 2. List of antibodies used for immunofluorescence, western blot and *in situ* hybridization experiments.**

| Antigen | Immunogen | Manufacturer | Application | Dilution |
|---|---|---|---|---|
| GAPDH | Glyceraldehyde 3-phosphate dehydrogenase (GAPDH) is an ubiquitous glycolytic enzyme present in reasonably high levels in almost all tissues. | Millipore, (Cat. #ABS16, rabbit monoclonal | WB | 1:2000 |
| BDNF | Synthetic peptide within Human BDNF aa 150 to the C-terminus. The exact sequence is proprietary. | Abcam, (Cat. #ab108319), rabbit monoclonal | WB | 1:1000 |
| | | | IF | 1:200 |
| IDO1 | KLH-conjugated linear peptide corresponding to the N-terminal region of mouse IDO-1. | Millipore, (Cat. #MABF850), mouse monoclonal | WB | 1:1000 |
| | | | IF | 1:100 |
| TPH2 | Tryptophan hydroxylase (TPH) is the rate-limiting enzyme in the biosynthesis of serotonin by converting tryptophan to 5-hydroxy-L tryptophan. TPH-2 is restricted to neuronal cells and the central nervous system. | CST, (Cat. #51124), rabbit monoclonal | IF | 1:200 |
| Nestin | Nestin is expressed in both mature and precursor neuronal and glial cells, as well as in the developing brain and in the brain and spinal cord following damage, Nestin is widely accepted as a marker of neural stem/progenitor cells. | CST, (Cat. #4760), Mouse monoclonal | IF | 1:400 |
| DCX | Doublecortin is a microtubule associated protein that stabilizes and bundles microtubules. | CST, (Cat. #4604), rabbit monoclonal | IF | 1:300 |
